# HmtDB, a Human Mitochondrial Genomic Resource Based on Variability Studies Supporting Population Genetics and Biomedical Research

**DOI:** 10.1186/1471-2105-6-S4-S4

**Published:** 2005-12-01

**Authors:** Marcella Attimonelli, Matteo Accetturo, Monica Santamaria, Daniela Lascaro, Gaetano Scioscia, Graziano Pappadà, Luigi Russo, Luigi Zanchetta, Mila Tommaseo-Ponzetta

**Affiliations:** 1 Dipartimento di Biochimica e Biologia Molecolare, Università di Bari, Via E. Orabona 4, 70126 Bari (Italy); 2 Java Technology Center, IBM Semea Sud, Via Tridente 42/14, 70125 Bari (Italy); 3 Dipartimento di Zoologia, Università di Bari, Via E. Orabona 4, 70126 Bari (Italy)

## Abstract

**Background:**

Population genetics studies based on the analysis of mtDNA and mitochondrial disease studies have produced a huge quantity of sequence data and related information. These data are at present worldwide distributed in differently organised databases and web sites not well integrated among them. Moreover it is not generally possible for the user to submit and contemporarily analyse its own data comparing them with the content of a given database, both for population genetics and mitochondrial disease data.

**Results:**

HmtDB is a well-integrated web-based human mitochondrial bioinformatic resource aimed at supporting population genetics and mitochondrial disease studies, thanks to a new approach based on site-specific nucleotide and aminoacid variability estimation. HmtDB consists of a database of Human Mitochondrial Genomes, annotated with population data, and a set of bioinformatic tools, able to produce site-specific variability data and to automatically characterize newly sequenced human mitochondrial genomes. A query system for the retrieval of genomes and a web submission tool for the annotation of new genomes have been designed and will soon be implemented. The first release contains 1255 fully annotated human mitochondrial genomes. Nucleotide site-specific variability data and multialigned genomes can be downloaded. Intra-human and inter-species aminoacid variability data estimated on the 13 coding for proteins genes of the 1255 human genomes and 60 mammalian species are also available. HmtDB is freely available, upon registration, at .

**Conclusion:**

The HmtDB project will contribute towards completing and/or refining haplogroup classification and revealing the real pathogenic potential of mitochondrial mutations, on the basis of variability estimation.

## Background

Thanks to its outstanding features, mitochondrial DNA (mtDNA) has been widely exploited both in population genetics and mitochondrial disease studies. In particular, the high mutation rate, absence of recombination, and maternal transmission make this molecule suitable for evolutionary studies aimed at tracing the migrations which led to the colonization of the various geographic areas of the world. Nevertheless, mitochondrial DNA also plays an important role in the oxidative metabolism of the cell, as it encodes for different subunits of the proteins involved in the respiratory chain. Hence, mutations occurring in mitochondrial DNA can alter the oxidative phosphorylation which seriously damages cells and tissues.

Mitochondrial DNA is a haploid molecule, and the number of identical copies of mitochondrial DNA per human cell ranges from 10000 to 100000 molecules. However, due to an inefficient repair system, mitochondrial DNA mutations are more easily fixed with respect to nuclear DNA, thus making mtDNA transitorily heteroplasmic (i.e., different molecules of mtDNA may be present in the same cell: wild-type and mutated). Indeed the mtDNA population rapidly becomes homoplasmic being the mutation fixed in one individual. If this mutation is not apparently deleterious and is transmitted to the following generations, then this mutation can be considered fixed in the lineage.

The mtDNA genome of two unrelated individuals may differ by about 50 nucleotides [1,2]. However, the number of mitochondrial Single Nucleotide Polymorphisms (mtSNPs) between two unrelated individuals is proportional to their divergence time. Study of these polymorphisms in various human populations has allowed to group differing human mtDNAs in haplogroups, each containing a subset of mtDNA sharing characteristic mutations acquired from the same ancestral mtDNA molecule. Hence, various population lineages may be described by means of a phylogenetic network, in which the top nodes define haplogroups and the tips haplotypes [3]. Major haplogroups are ethnic-specific, and their classification is becoming increasingly well defined with the growth in the type and quantity of molecular data available.

Mitochondrial disorders – associated with dysfunctions of the Oxidative Phosphorylation (OXPHOS) system – are caused by genetic defects both in the mitochondrial and nuclear genome, leading to energy metabolism errors, and have an estimated frequency of 1 out of 10000 live births. Due to the important role played by the OXPHOS system in ATP production, the causes and effects of mitochondrial disorders are extremely heterogeneous and complex. This explains the pressing need for further research on this topic, despite the many studies on mitochondrial disorders published in the last 20 years.

Population genetics studies based on analysis of mtDNA and mitochondrial disease studies have produced a huge quantity of sequence data and related information. Classified as RFLPs, mtDNA SNPs, pathogenic mutations, HVS1 and HVS2 sequences, and complete mtDNA sequences, these data are now distributed worldwide in variously organized databases and web sites, not all well integrated. Several specialized mitochondrial databases for human and primates have been designed and implemented, providing good quantity and quality of human mitochondrial data: MITOMAP [4], HVRBASE [5], mtSNPs [6] and mtDB (Ingmann et al, ). The two last databases report frequency data associated with mitochondrial SNPs, whereas MITOMAP simply associates mtSNP with the different phenotypes and to literature data.

HmtDB, the resource presented here, is aimed at filling the gap, gathering all the complete human mitochondrial genomes worldwide distributed and enriching sequence information with statistically validated variability data estimated through the application of specific algorithms implemented in an automatically running Variability Generation Work Flow (VGWF). A second work flow, called Classification Work Flow (CWF) is implemented in the resource to perform automatic classification of newly sequenced genomes. Here we describe in detail HmtDB aims and design as well as its potential applications and future developments.

## Results

### HmtDB Aims and design

The aims of HmtDB are (1) to collect and integrate all human mitochondrial genomes publicly available, (2) to produce and provide the scientific community with site-specific nucleotide and aminoacid variability data estimated on all available human mitochondrial genome sequences through the automatic application of VGWF, (3) to allow researchers to analyse their own human mitochondrial sequences (both complete or partial) in order to detect automatically any nucleotide variants according to the revised Cambridge Reference Sequence (rCRS) and to predict their haplogroup paternity. This is automatically performed thanks to the application of the CWF.

VGWF is applied both to the entire HmtDB genome collection and to continent-specific subgroups. The resulting site-specific variability values highlight functional constraints in both control and coding regions and, thus, distinguish polymorphic sites from constrained ones. From a population genetics point of view, this allows complete and/or refined mitochondrial haplogroup classification, providing a more precise interpretation of population sequence data and thus a refinement of phylogenetic models. From a clinical point of view, it contributes towards shedding light on the real pathogenic potential of certain mitochondrial mutations, not yet clearly associated with any mitochondrial pathology.

#### Architecture and macro-functions

HmtDB is a bioinformatic platform which allows storage, query and analysis of human mitochondrial sequences. It is available at the web address  through a log-in procedure, after free registration.

This web-based application is organized as a relational database, storing complete human mitochondrial genomes, data related to the sample and the subject from which the mtDNA was extracted, and the results of variability analyses performed through the automatically running VGWF implemented in the resource itself. A web-service interface to the PubMed public database also allows automatic retrieval of reference information during sequence annotation.

**Figure 1:**
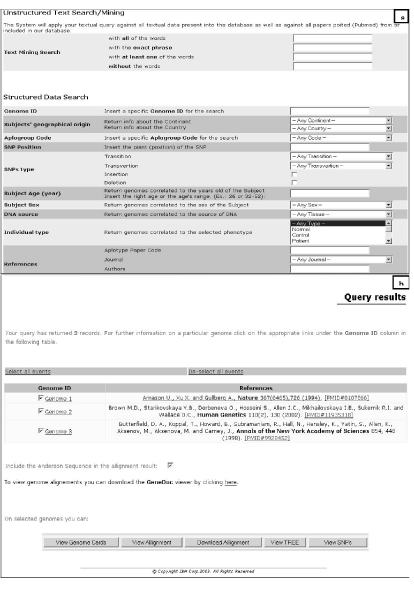
**HmtDB query pages. **Design of pages related to Query function as it will be implemented in the database. a) Multicriterion query form. b) Example of a query result.

Five macro-functions have been designed for HmtDB and are available to users after they have completed the log-in procedure. From the first page, users can (1) browse through the contents of the database, in order to view and download multialigned sequence data and site-specific nucleotide variability data, both regarding the entire database and continent-specific subsets; (2) analyse their own mitochondrial sequences in order to classify them automatically according to the latest mitochondrial haplogroup classification; (3) view the history of previously performed analyses and, if necessary, recover them, thanks to the existence of a personal session for each user (this is why users must register their access to HmtDB); (4) query the database through (a) a simple text search form or (b) a multicriterion form made up of pop-up menus and free text retrieval windows; (5) submit a new human mitochondrial genome through an easy-to-fill-in web interface. Functions 4 and 5 have been designed but are not yet implemented. Figures 1a and 1b show the design of the "Query" pages, as they will appear and implemented.

#### Structure and content of database

**Figure 2:**
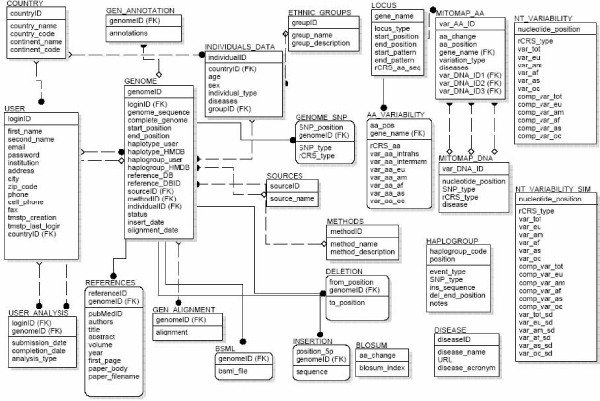
**HmtDB relational database. **HmtDB relational structure includes 24 tables, storing complete set of HmtDB data linked to each other.

The HmtDB database is of relational type, designed and implemented using the DB2 IBM Database Management System. Currently, it is composed of 24 tables (Figure 2) which store human mitochondrial genome sequences and their characteristic data such as geographic, ethnic and/or population and linguistic information, as well as information about the source from which the DNA was extracted and the method used to extract and sequence it. The 24 tables also include data tables for observed and simulated site-specific nucleotide and aminoacid variability calculated through HmtDB, haplogroup classification, mitochondrial aminoacid similarity values obtained from the mtREV substitution matrix [7], users, and publications. Finally tables reporting data from the MITOMAP database about nucleotide and aminoacid mitochondrial polymorphisms and pathological mutations are part of the database.

At present, 1255 human mitochondrial sequences are stored in the database. Each entry is represented by a human mitochondrial sequence, and can be viewed through its Genome Card. The information associated with each Genome Card is grouped in two categories: manually annotated data derived from source databases (see section on "DATA SOURCE") and data produced by the application of nucleotide and aminoacid site variability software ("HmtDB WORKFLOWS"). In the first category, the associated information is: geographic origin of the subject from whom the mtDNA comes, sex, age, disease acronym (if the genome sequence is part of a mitochondrial disease study), biological source of mtDNA, bibliographic references, haplogroup and haplotype code as reported from the authors, and the sequencing protocol adopted. These are subdivided into several tables, e.g., GENOME, REFERENCES, INDIVIDUAL DATA, COUNTRY, ETHNIC GROUPS, etc.. Missing information are left empty.

Data in the second category are mtSNPs, deletions and/or insertions determined by comparing each genome with the revised Cambridge Reference Sequence [8,9]. Nucleotide and aminoacid site-specific variability values are also associated with each mtSNP. The aminoacid inter-mammalian site-specific variability values estimated for the 13 mtCDS from 60 mammal species whose mitochondrial genome has been completely sequenced are also annotated. These data are structured in the tables entitled NT_VARIABILITY, NT_VARIABILITY_SIM (for simulated data: see VGWF section) and AA_VARIABILITY.

Moreover information about mtSNPs with phenotypic effect, derived from MITOMAP database annotations (MITOMAP_DNA and MITOMAP_AA tables in Figure 2), are linked to information on mtSNPs presented by each single mitochondrial sequence, and shown on the relative Genome Card. This constitute a good example of how a relational database structure can link various types of data coming from different sources.

Mitochondrial haplogroup classification is also stored in the database (HAPLOGROUP table in Figure 2). It is a summary of human mitochondrial haplogroup data available in the literature, revised by A.Torroni. For each haplogroup, the code and set of mtSNPs defining it are annotated in the HAPLOGROUP table. In this way, the VGWF can predict the haplogroup paternity of each genome, shown on the Genome Card.

Due to the existence of "personal sessions" for users accessing HmtDB, tables USER and USER ANALYSIS have also been incorporated in its structure.

#### Web interfaces

Web interfaces for database query, analyses, query results and analysis results, submission and personal sessions have all been developed using Java Server Pages (JSP). JSPs are more efficient, easier to use, more powerful and more portable than traditional CGI. They have been extensively linked to each other, so that users can easily access data independently from the navigation starting point. A complete set of "help" information, widely distributed throughout each web page, supports users during navigation and interpretation of data derived from the application of the HmtDB VGWF to human mitochondrial genomes.

The "Welcome" page is the first page of HmtDB. It gives some general information about the resource, and offers the possibility of registering (through an appropriate form) or of logging in directly to HmtDB (provided that a previously successful registration has been completed).

After log-in, users access a main menu page, from which HmtDB macro-functions are available.

The HmtDB browsing function allows the selection of a specific genome through a menu reporting the list of the database source Accession Numbers, displaying the Genome Card of the selected genome. This is the page on which most of the results available through HmtDB are shown: both CWF and VGWF analytical results are displayed, together with information from MITOMAP and mtREV mitochondrial substitution indices [7] associated with each mitochondrial aminoacid SNP detected in each annotated sequence. It is also possible to access, through many links, continent-specific nucleotide and aminoacid site variability values, and simulated site-specific nucleotide variability values. Help information about these results can also be obtained by mouse moves. A download section, providing the multialignment of the entire contents of the database, multialignments for continent-specific subsets, and site-specific variability data, is also available in the browsing function.

The "Analysis" page offers the possibility of inserting a human mitochondrial sequence and analysing it according to the CWF procedure. Results are shown through the generation of a new Genome Card on which the relative haplogroup classification is displayed, as well as the VGWF results on mitochondrial nucleotide variants detected for that sequence.

The "Query" and "Submission" pages have not yet been implemented, and the words "work in progress" highlight this condition. However, it is worth mentioning that, if a new human mitochondrial sequence is submitted, HmtDB automatically checks that it is not already present in the database, and in any case the new sequence is analysed according to the CWF procedure.

Lastly, the "My HmtDB Sessions" page displays the list of analyses performed by each user, together with the date and completion status (marked by different colours).

## Methods

### Data source

The first bulk of data stored in the database consists of 1255 human mitochondrial sequences, of which 560 are coding region sequences only, and the rest are complete genomes. They were retrieved from GenBank , EMBL , MITOMAP , GOBASE , Mitokor  and mtDB . The total resulting dataset is composed of 2291 genomes analysed with the CleanUP program [10] in order to detect redundancies automatically. CleanUP software is periodically applied to the entire set of human mitochondrial genomes publicly available, searching for sequences with 100% similarity and 100% overlaps. A screen of the CleanUP results distinguishes identical sequences derived from the same individual (artificial redundancy due to the presence of the same sequence in different databases) from identical sequences derived from different individuals (biological redundancy). Only biological redundancies were recovered, yielding the present dataset of 1255 sequences, corresponding to 1006 haplotypes, at present stored in HmtDB. The number of haplotypes is the number of non-redundant sequences obtained through the CleanUP application. Future updates will be carried out periodically, partly relying on authors' direct submissions.

Information on the annotation of genomes in HmtDB is derived from the entry of the database from which the sequences were retrieved. Further data such as geographic origin, age, sex, haplogroup, disease acronym, tissue, and sequencing method were extracted from the relative literature. Ethnic information was annotated following the Ethnologue database .

Quality of the sequences stored in HmtDB is under the responsibility of the sequence producers and of the source databases. Anyway in case of personal submissions, a check for possible errors will be made before the submission procedure is complete.

Inter-species aminoacid site variability was estimated on the multialignment of the 13 mtCDS of 60 complete mitochondrial genomes from various mammal species. These were retrieved from the AMmtDB database [11].

Information about polymorphic and pathological mutations was retrieved from MITOMAP and is organized in tables describing the phenotypic effect of each mutation.

### HmtDB workflows

**Figure 3:**
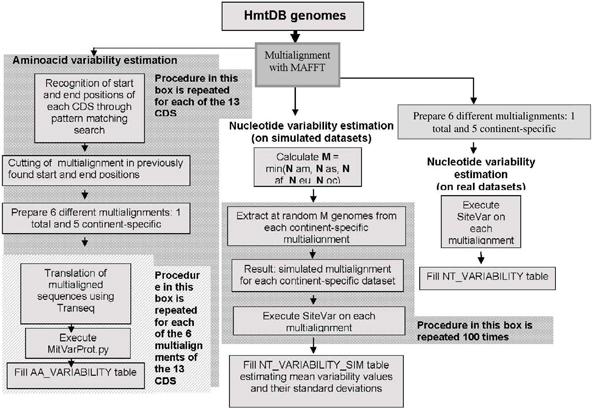
**Variability Generation Work Flow (VGWF)**. Starting from the multialignment of all the genomes stored in the database and of continent-specific subsets performed with MAFFT program, three variability analyses are executed separately: nucleotide variability values for both the entire set of genomes and the continent-specific subsets are estimated through SiteVar program applied on real dataset (right hand side of the Work Flow) and on 100 simulated multialignments obtained through an automatic procedure for each continent-specific genomes dataset (in the middle of the Work Flow); aminoacid variability data (left hand side of the Work Flow) are produced by applying MitVarProt software to the 13 multialigned mitochondrial protein coding genes, automatically selected from the entire starting nucleotide multialignment and translated into aminoacid sequences through TRANSEQ program; the data produced through this three procedures are inserted in NT_VARIABILITY, NT_VARIABILITY_SIM and AA_VARIABILITY tables of the relational HmtDB structure respectively.

The execution of both CWF and VGWF procedures requires the application of a set of bioinformatics methods described below. Figure 3 reports the VGWF flow chart. The first step of both procedures is alignment of the human mitochondrial genomes. In the case of CWF, this is performed between the sequence to be analysed and the rCRS sequence; in the case of VGWF, it is performed on all the contents of the database and on continent-specific subsets. In both cases, multialignment is carried out by executing the MAFFT program [12], a multiple sequence alignment program suitable for a great number of long sequences, based on fast Fourier transform, which allows rapid detection of similar segments. Nucleotide and aminoacid variability data are produced by applying SiteVar [13] and SiteVarProt [14] software, respectively, to the multialigned genomes. The versions implemented in HmtDB are revised versions developed in collaboration with David Horner, with the aim of adapting the software to analysis of mitochondrial sequences. In particular, the SiteVar algorithm has been improved by assigning different scores to transition and transversion and by considering gapped sites. In particular, the scores now considered are: 1 for each transition, 2 for each transversion in the site, and 2 for a gapped site. The SiteVarProt program is transformed into MitVarProt, in which the Blosum-like index is replaced with indices derived from the mtRev substitution matrix [7] based on multialigned mitochondrial codified proteins, and a score for gapped sites has also been introduced. The 13 mitochondrial coding for the protein genes (mtCDS) of the human mitochondrial genome are automatically selected from the entire nucleotide multialignment stored in the database, by means of pattern matching. Translation into aminoacid sequences is then executed by applying the TRANSEQ program (EMBOSS package) on the single gene-specific multialignment. The resulting aminoacid multialignments are submitted to site-specific variability analyses through the MitVarProt software.

#### CWF

CWF procedure is performed on a single human mitochondrial sequence (Query), and aims at predicting its haplogroup paternity. It consists essentially of an automatic comparison between Query and rCRS sequences, with the aim of detecting the pattern of Query mtSNPs. Matching an obtained pattern against the haplogroup classification stored in HmtDB can predict the Query sequence haplogroup paternity. A Genome Card of the analysed genome is generated and displayed, on which haplogroup paternity is expressed as a list of haplogroups for which a match was found, and a percentage value of the detected variations with respect to the total number of variations defining the haplogroup. Also in this case, a complete set of links, supported by help information, allows users to navigate through the data and to retrieve information about haplogroups.

#### VGWF

VGWF is performed both on the entire contents of the database, and on continent-specific subsets. It aims at estimating nucleotide and aminoacid site-specific variability through the application of appropriate software to multialigned data. Also in this case, the procedure is completely automatic (including division of the entire dataset into continent-specific subsets) and is repeated each time the contents of the database is significantly updated, as the site-specific variability software performs statistical estimates whose results change significantly only in cases of considerable changes in the starting data (number of sequences). In order to make these results statistically more significant, bootstrap values are also estimated. This is done by an automatic simulated sampling procedure which, starting from real data stored in the database, estimates a number M of sequences equal to the number of the less numerous continent-specific dataset. Then M sequences from each continent-specific dataset and from the entire database are chosen at random 100 times, and 100 site-specific nucleotide variability values are estimated for each subset on these simulated samples. The final result, consisting of the mean site-specific variability value plus its standard deviation, estimated for each dataset on the 100 simulated values, is then displayed in appropriately formatted tables, and can also be downloaded. The simulated variability values statistically support real ones, as they provide an idea of the statistical significance of the dimension of the real samples: the closer the simulated value is to the real value, the more "ideal" is the dimension of the real sample stored in the database.

## Conclusion

Progress in sequencing techniques is making the sequencing of complete genomes increasingly easy, in terms of human resources, so that since 2000 the number of complete genomes has significantly increased [3,15-26], paving the way for "mitochondrial population genomics" [27,28]. This new era opens up new perspectives both in population genetics and mitochondrial disease studies, especially when new approaches, such as site-specific variability estimation, are followed.

The HmtDB project is intended to store and analyse human mitochondrial genomes in a well-integrated web-based bioinformatic resource, in order to support population genetics and mitochondrial disease studies, thanks to a new approach based on site-specific nucleotide and aminoacid variability estimation. This will contribute towards completing and/or refining haplogroup classification on the basis of variability values obtained from HmtDB, in order to detect new haplogroups and/or modifying existing classifications. In addition, site-specific variability may reveal the real pathogenic potential of mitochondrial mutations, excluding those which present too high inter-and intra-species nucleotide and aminoacid variability values (indicative of low functional constraints in the region presenting them), and focusing attention on those mutations presenting more intriguing variability values.

In this way, HmtDB may contribute in a more rigorous way to quantitative estimation of the pathogenetic proneness of mutated sites and to the detection of ethnic-specific sites, and consequently to refining haplogroups.
